# Objective Structured Clinical Examinations (OSCEs), psychiatry and the Clinical assessment of Skills and Competencies (CASC) Same Evidence, Different Judgement

**DOI:** 10.1186/1471-244X-11-85

**Published:** 2011-05-16

**Authors:** Steven Marwaha

**Affiliations:** 1 Rm B149. Medical School Building, Gibbet Hill Campus Warwick Medical School University of Warwick Coventry CV47AL UK

## Abstract

**Background:**

The Objective Structured Clinical Examination (OSCE), originally developed in the 1970's, has been hailed as the "gold standard" of clinical assessments for medical students and is used within medical schools throughout the world. The Clinical assessment of Skills and Competencies (CASC) is an OSCE used as a clinical examination gateway, granting access to becoming a senior Psychiatrist in the UK.

**Discussion:**

Van der Vleuten's utility model is used to examine the CASC from the viewpoint of a senior psychiatrist. Reliability may be equivalent to more traditional examinations. Whilst the CASC is likely to have content validity, other forms of validity are untested and authenticity is poor. Educational impact has the potential to change facets of psychiatric professionalism and influence future patient care. There are doubts about acceptability from candidates and more senior psychiatrists.

**Summary:**

Whilst OSCEs may be the best choice for medical student examinations, their use in post graduate psychiatric examination in the UK is subject to challenge on the grounds of validity, authenticity and educational impact.

## Background

The Objective Structured Clinical Examination (OSCE), originally developed in the 1970's, has been hailed as the "gold standard" of clinical assessments for medical students [1] and is used within medical schools throughout the world [2]. In terms of Miller's triangle [3] describing a framework for clinical assessment, OSCEs aim to examine skills and ability at the "shows how" level, with an expectation that this might reflect performance in day to day real life clinical situations.

OSCEs developed in response to the difficulties identified with traditional long case clinical examinations. There was very often a lack of transparency about the objectives of the assessment and the competencies required to succeed. Also, no clear marking system resulted in variability between assessors and individual examiners were not always consistent over time [4]. This subjectivity, the potential for examiner bias [5], and the use of small numbers of real cases was also linked to perceived unfairness.

Thus the charge was that long cases lacked reliability and validity and were unfair to those assessed. In his seminal paper on OSCEs, Harden [6] outlined an alternative, espousing its objectivity, its reliability and the controlled standardized testing of multiple competencies, thereby eliminating non-candidate variance in results.

This debate paper aims to critically appraise the use of OSCEs as a method of assessment in the membership examination of the Royal College of Psychiatrists UK (MRCPsych), although the arguments are likely to apply to other postgraduate psychiatric examinations. The OSCE in the MRCPsych is called the Clinical Assessment of Skills and Competencies (CASC) and it seeks to measure psychiatric competence. The framework of Van der Vleuten's utility model [7] is used to examine the CASC's strengths and weaknesses from the position of a senior psychiatrist, seeking to promote psychiatric skills and ultimately to sustain improving patient care. Balancing the different elements of reliability, validity, acceptability, educational impact and costs, and the needs of stakeholders in reaching a compromise is inherent to the model [8]. I examine evidence pertaining to OSCEs in general before specifically discussing the CASC.

## Discussion

### Reliability

Many researchers have studied the reliability of OSCEs in a wide variety of subjects, most frequently focussing on their use with medical students. Large and well conducted investigations show that OSCEs tend to be reliable [9]. Generalisibility coefficients seem however to have a fairly wide range from 0.4 to 0.85, with the bulk of coefficients being between 0.5-0.6 suggesting moderate reliability [10]. This variability is likely to be due to examinees variable performance on different OSCE stations (content specificity) but means that many OSCEs, including high stakes examinations do not reach the reliability coefficient threshold of 0.8 or over [11] which is widely regarded as the marker of sufficiency.

Whilst reliability will be improved by increased sampling of content [12], a variety of other factors such as the number of stations required and thus time taken may have an influence also. For example in an OSCE assessing surgical residents, high reliability (>0.8) was dependent on using 38 stations and a 3 hour test [13], raising questions of the assessment depth when each station lasted for 4.5 minutes. A solution to the potential superficiality of stations whilst maintaining reliability is to lower station number to 8 but increasing test length to 4 hours [14]

OSCEs usually require at least 4 hours of testing for them to be reliable overall [15]. With such long examination times, concerns about costs and acceptability to students are real issues. Also difficulties with organisation and examinee tiredness begin to ultimately affect the psychometric properties of the assessment. This may be particularly difficult to justify in high stakes exams such as the CASC.

Another issue is that whilst increasing test length may increase the reliability of assessment, it appears to do so differentially for the range of competencies under test. Thus for communication skills, test time only needs to be 2 hours to achieve a coefficient of 0.7, but 6 hours is required for reliable assessment of history taking skills [16]. With the CASC the Royal College of Psychiatrists seek to assess history taking skills, mental state tests and more complex process based ability all in one assessment. It is unclear whether it is feasible to test such variable content in a through way and with good reliability.

The CASC like many other OSCEs replaced long case examinations with the express purpose that it would have better reliability. However it is not at all clear whether OSCEs are necessarily more reliable than traditional long cases. Wass et al [17] report a well constructed and naturalistic experiment with final year medical students undergoing OSCEs, with a subsample sitting observed and unstandardized long cases. With an assessment of 3.5 hours for history taking, long cases were equally reliable as OSCEs. A review of the available evidence suggests that from 1-4 hours of testing time, an examination based on long cases is at least as reliable as one based on OSCEs [8].

Assessment objectification or "a set of strategies designed to reduce measurement error" [18] is a major part of the value placed on OSCEs and on the CASC. However reliability does not wholly depend on objectification and standardizing the testing environment. Appraisal of a number of small studies [8] suggests that it is sampling across a number of clinical domains that reduces this measurement error as opposed to attempts at objectivity per se. The CASC in fact implicitly accepts some degree of subjectivity in assessment by using a global score to assign pass/fail decisions, implemented because global judgments of mastery appear to be more reliable than checklists [19].

There is no published data on the reliability of the CASC. Given the number of stations and testing time, the reliability may well be reasonable but this remains to be seen. The caveats about the reliability of OSCEs (in general and in comparison to long cases), raise the question of whether the CASC is the ideal response to the perceived difficulties of using long cases in the previous MRCPsych system.

### Validity

There are numerous studies that have investigated the validity of the use of OSCEs in clinical examinations of medical students and a full review of all of these is outside the scope of the current discussion. In the main, by demonstrating that the results of OSCEs: relate to other examinations; discriminate between candidates of different experience; and that on the face of it the exam covers appropriate areas, these studies indicate OSCEs can have face, content, construct and concurrent validity. Investigations spanning the globe and multiple sub-specialities with undergraduates show similar results, thereby increasing confidence [7,20].

For example in Jamaica, paediatrics students taking an OSCE found it to have a high level of fairness, authenticity and comprehensiveness suggesting face and content validity [21]. In a large study of a cohort of medical students (N = 435) comparisons were made between scores on an OSCE and those from work place based assessments, multiple choice exams and essays. There was a high degree of concurrent validity with correlation coefficients reaching 0.7 in most of the measurements [22].

Alternative views and contradictory evidence on the validity of OSCEs is however also available. For example an investigation in Canada of concurrent validity of OSCE test scores with other assessment procedures found correlation coefficients ranging from 0.1 to 1 with the coefficient rising to above 0.7 only in a minority of the comparisons [10]. In a selective narrative review of the OSCE literature, Barman [23] is highly critical of the validity of OSCEs, suggesting that their predictive and concurrent validity is, in general, too low to be useful and that cognitive tests are more "psychometrically efficient" as measures of performance. He concludes that OSCEs should be one of a number of different examinations to test clinical skills.

Concerns were raised early in the OSCE movement about whether they could capture and adequately reflect the complexity of psychiatry cases within medical student exams. Hodges completed a number of investigations in this area. By examining the performance of 33 students and 17 Residents, Hodges et al [24] reported that the Psychiatry OSCE had construct and concurrent validity. These results were repeated in a later, methodologically robust and much larger sampled investigation [25]. Medical students have also found Psychiatry OSCEs to be acceptable and feasible [26].

Thus it does appear that for medical students, an OSCE approach to examining Psychiatry cases can be valid. It is much less clear whether an OSCE such as the CASC can assess higher order thinking or advanced psychiatric skills that a senior Psychiatrist would need to have. Hodges investigated the suitability of OSCEs to examine Psychiatry Residents whilst validating an OSCE for medical students. The Residents believed the OSCE would enable the identification of inadequate or unsafe medical students, but were disparaging about whether they were suitable to assess aspects of Psychiatry such as "interpersonal connection", transference issues or other complex phenomenon that are clinically important [27].

Thus a major area of concern of using OSCEs such as the CASC in postgraduate psychiatric assessment is the authenticity of clinical encounters. One aspect of this is the validity of using actors or standardized patients in psychiatry examinations, although the available evidence is fairly reassuring. Studies from general medicine suggest that doctors cannot pick out standardized patients played by actors [28,29].

Whilst nearly all of the literature regarding psychiatric simulated patients is descriptive it does suggest face validity. The only psychometric assessment to the author's knowledge pertains to a simulated depression patient acted over the course of 1 year and this suggested a high degree of reliability [30]. Despite this it is clear that actors will not be able to emulate signs such as thought disorder, blunted affect or disorganization. Also risks remain that simulated patients will represent a text book as opposed to a real life case.

Another aspect of authenticity is the extent to which simulations really do represent real psychiatric encounters. Stations of 10 minutes or less within the CASC inevitably mean that small component parts of psychiatric skills will be tested and a holistic assessment of the whole person is unlikely to be possible [31]. Underlying the OSCE method is a reductionist paradigm suggesting human behaviour and problem solving skills can be split into component parts and then meaningfully scrutinised. Hodges [32] eloquently argues that the validity of an assessment is intricately linked to and a function of context. It is therefore doubtful that a single or a number of 10 minute OSCE stations can represent the depth of a 1 hour clinical assessment with a single patient, something which is fairly routine in clinical practice.

CASC stations are task driven and difficult to generalize. They would seem very distinct from real life clinical situations which are much more about process and linking several aspects of the history to produce a formulation. Indeed assessing ability within a CASC, to do a psychodynamic formulation would seem somewhat impossible. Even Harden [6] in his original paper on OSCEs suggested that compartmentalisation of knowledge and discouraging people from looking broadly at difficulties was a major issue for OSCEs. Interestingly Harden advocated additional testing using a long case or by some form of work based assessment when OSCEs were used.

It has been asserted that OSCEs such as the CASC risk sacrificing validity for objectivity [33] with the potential to test complex processing and judgment skills becoming subservient to the needs of standardization. The CASC like other OSCE values thoroughness, by requiring candidates to complete numerous component tasks within the competency being tested. However senior psychiatric clinicians are not necessarily thorough, but are generally accurate at quickly identifying the salient difficulty and features of a patient [34].

Testing context can significantly predict performance in an OSCE [35]. Therefore focussing a candidate on a particular area at a CASC station may in itself reduce the validity of the assessment. Such direction does not usually happen for senior clinicians who need to work out what information is salient from what the patient has said thus far. Arguably that is the meta-skill, which lies in getting order out of chaos.

The Royal College of Psychiatrists have used a blueprinting method [36] in order to develop and demonstrate content validity [37] of the CASC. However there are no published studies of the construct and predictive validity for the CASC. As is the case with reliability, global judgements appear to show better concurrent validity than checklists in surgery [19,38] as well as in Psychiatry [39]. As a result of this the CASC uses global judgements in its marking scheme. Whilst this increases the flexibility given to the examiners, this mirrors the flexibility in judgements that examiners of long cases had [26] that were criticized on the basis of fairness.

Underlying these difficulties of validity and the CASC is a lack of clarity about what would constitute a valid clinical examination for those seeking to become Consultant Psychiatrists. In other words, there is no "gold standard" by which other assessments could be compared.

Considering alternatives to the CASC, work place based assessments might offer a partial alternative. However as they currently stand, they suffer their own problems of subjectivity with those assessed always being known to assessors and assessor bias being highly likely after a 4-6 months period. Gleeson [40] describes a process of making the long case more objective and able to validly assess ability. Whilst he spells out a compelling argument for the Objective Structured Long Examination Record no psychometric data is presented although it does appear to increase authenticity.

### Educational impact

According to Van der Vleuten [7], "assessment drives learning though its format". In the case of OSCEs and more specifically the CASC, this may have a range of consequences, some desirable and others less clearly helpful. These impacts can be at the level of learners and at the level of the profession, with effects at the latter level potentially affecting patient care.

One example of a positive educational impact of OSCE use is that medical student performance improved and teaching methods became more standardized at a US medical school [41]. Using OSCEs can also result in students spending much more time on wards [42] than previously. There is a risk however, that medical students will learn checklists used in OSCEs resulting in a reduction in their skills [43].

Given assessment objectives should reflect educational objectives the key question of educational impact is whether the CASC will or can drive learning and skills that are needed to be an effective senior Psychiatrist. It is clear that the content of OSCEs can influence candidates learning before and long after the test [1].

Therefore one effect of the CASC could be that it drives a diffusion of skills which are more generalist in nature than specialist [35]. Students organize their learning around a test. As such if passing the CASC means practicing tasks that could be asked in 10 minutes, why bother developing interview, assessment, formulation and management skills which are more complex.

The results of this may be far reaching and difficult to predict. The CASC format may determine what features in Consultants are most highly valued and thus fashion facets of medical professionalism. The CASC may also subtly shift the skill set of senior psychiatric clinicians towards a compartmentalising approach potentially reducing the depth of clinical knowledge and its uses. This is despite the National Health Service, the major employer in the UK demanding Consultant Psychiatrists focus on the most complex of patients [44].

### Cost

Clinical examinations tend to be costly because of the amount of examiner or patient time needed as well as indirect costs. Experts in the field regard OSCEs to be expensive [45], possibly because to achieve the claimed reliabilities many stations are needed and testing times long.

However direct comparisons of OSCEs with other methods such as long cases are limited and the cost balance of OSCEs may even be subject specific. In Psychiatry using an OSCE compared to a long case for medical students was found to involve less faculty hours and therefore led to savings [46]. In surgery, in comparison to a structured oral examination, an OSCE was more costly [47].

### Acceptability

Given that OSCEs seek to deliver objectivity and transparency in the assessment process, this to some extent explains why they are acceptable to medical students [21] and to qualified doctors [48].

However two investigations do seem to suggest that for Psychiatry at least, seniority predicts having doubts about the value of OSCEs. In the US, Psychiatry Residents were more cautious about the usefulness of OSCEs to test higher psychiatric skills than to test the safety of medical students [27]. Secondly in an intriguing, but albeit small (N = 18) study of participants who attended a CASC revision course in the UK, approximately 70% of attendees did not agree that there was "no longer a need to use real patients in post-graduate clinical exams". In comparison to the previous examination system, whilst half preferred the CASC, half were undecided or wished to have a return to using long cases [49].

It is unclear whether the profession itself finds the CASC or the types of Psychiatrists it produces acceptable or not as there is no available literature. Clinicians' views about how they themselves were assessed are likely to affect how they perceive the utility of the CASC. Whilst reservations may represent a generational effect, it would seem inappropriate to exclude any dissenting voices as "old fashioned" and protagonists of the CASC as "modern".

## Summary

OSCEs appear to show reasonable psychometric properties in terms of reliability and validity when tested in specific situations. The strongest evidence for their usefulness appears to be when they are an assessment method for medical students, where the overriding need is to prove safety. The case for their use in the MRCPsych appears to be more questionable given that they make tasks necessarily simpler than real life and may not be able to test higher psychiatric clinical skills. Whilst the CASC may be reliable (although as yet unproven), there may be significant problems related to validity, authenticity and acceptability, including in its costs.

These difficulties are critical given that a lack of scope to test more complex psychiatric reasoning in a way that a longer clinical assessment might, could affect the standing of the profession and the future care of patients. Because it is a test, which is the gateway to becoming a senior Psychiatrist, a different balance between the elements of the utility model may be necessary than is currently the case. Such a re-appraisal should give much higher credence to validity, and whilst respecting the needs to deliver a fair and reliable assessment, reject the absolute primacy of the needs of those being assessed. Alternatives to the CASC in the context of the MRCPsych should be investigated further. Despite the OSCEs ubiquity, one size does not fit all.

## Appendix A

The CASC is the only clinical examination in the membership examinations for the Royal College of Psychiatrists (MRCPsych). There are 16 stations with the pass mark being 12 out of the 16 stations. Two circuits are completed on the same day with the first circuit involving 8 stations lasting 7 minutes with 1 min prep time. The second circuit has 4 pairs of linked stations with each station lasting 10 minutes. This summative assessment is criterion referenced and needs to be passed to enable a candidate to enter higher specialist training which usually after 3 years leads to qualification as a Consultant. The CASC seeks to test competency in Psychiatry.

The CASC has replaced the previous system of part I and part II examinations which involved 2 long cases with each long case involving a partly observed interview of a real patient. Part II also involved an oral examination involving some standardized patient management problems.

## List of abbreviations

OSCE: Objective Structure Clinical Examination; CASC: Clinical Assessment of Skills and Competencies; MRCPsych: Membership of the Royal College of Psychiatrists UK

## Competing interests

The author declares that they have no competing interests.

## Authors' contributions

SM conceived of, investigated and wrote this article. The views expressed are the authors.

## Authors information

SM is Associate Clinical Professor of Psychiatry and a Consultant Psychiatrist in the Coventry Early Intervention Team for people with a first episode of psychosis. He has supervised and aided junior doctors sitting the CASC examination.

## Pre-publication history

The pre-publication history for this paper can be accessed here:

http://www.biomedcentral.com/1471-244X/11/85/prepub
